# Epi-endocardial asynchrony during atrial flutter followed by atrial fibrillation

**DOI:** 10.1016/j.hrcr.2021.01.001

**Published:** 2021-01-13

**Authors:** Lianne N. van Staveren, Frank R.N. van Schaagen, Natasja M.S. de Groot

**Affiliations:** ∗ Department of Cardiology, Erasmus Medical Center, Rotterdam, the Netherlands; † Department of Cardio-Thoracic surgery, Erasmus Medical Center, Rotterdam, the Netherlands

**Keywords:** Atrial flutter, Atrial fibrillation, Case report, Epi-endocardial asynchrony, Fractionation, Unipolar extracellular potentials

## Introduction

There is increasing evidence of an interrelationship between atrial flutter (AFL) and atrial fibrillation (AF). This is illustrated by the large proportion of patients with AF who also have AFL, and vice versa. Even after cavotricuspid isthmus ablation (CTI) for AFL, 23% of these patients develop AF^1^ and after AF ablative therapy, 20% of AF patients develop AFL.^2^

A possible explanation for the interaction between these 2 tachyarrhythmias is that they have similar triggers and substrates.^3^ For example, pulmonary vein ectopy may also trigger AF in patients with AFL. Remarkably, when solitary pulmonary vein isolation (PVI) was performed in patients with isolated AFL, AFL recurrence was reduced.^4^ Patients with AFL were randomized into PVI (n = 20), CTI (n = 23), or only antiarrhythmic drugs (n = 17), and interim analysis after 1.42 ± 0.83 years showed reduced tachyarrhythmia incidence in the PVI (10%) and CTI group (60.9%) compared to patients treated with antiarrhythmic drugs alone (82.4%, *P* < .001).

However, this observation was not confirmed in a meta-analysis.^3^ Nonetheless, a selection of AFL patients may thus benefit from an additional PVI but it is currently unknown which characteristics, besides common risk factors for AF (left atrial [LA] dilatation, age > 55 years, reduced left ventricular function), can be used to identify these patients. Thus, the interrelationship between AFL and AF seems only partly understood.

In this case report, we present a patient who has a typical, counter-clockwise AFL and develops AF after aortic valve replacement and surgical ablation of AFL. Intraoperative epicardial mapping of the atria showed elaborate regions with lines of conduction block (CB) and fractionated unipolar electrocardiograms (U-EGM). In these regions, multiple distinct waves propagating in deeper tissue layers were observed. Based on these observations, we postulate that increased endo-epicardial asynchrony (EEA) during AFL contributes to increased susceptibility to AF.

## Case report

A 77-year-old man with severe aortic stenosis and persistent AFL was admitted for elective aortic valve replacement and concomitant surgical AFL ablation. Written informed consent for cardiac mapping (MEC-2015-274) was obtained prior to surgery.

Before induced cardiac arrest, a mapping array of 128 electrodes (interelectrode distance: 2 mm) was used to cover the epicardial atrial surface of Bachmann's bundle (BB), the right atrium (RA), anterior LA including the left atrial appendage (LAA), and pulmonary vein area. In addition, recordings of the endocardial right atrial septum were obtained.

U-EGMs, sampled at a frequency of 1000 Hz, were automatically analyzed offline and manually checked using customized software. Local activation times were defined as the steepest negative deflection of U-EGM potentials and used to reconstruct wavemaps during 10 seconds of AFL.

Subsequently, distribution of AFL cycle length (AFL-CL) and AFL-CL variability were assessed at all locations. Variability of AFL-CL is defined as standard deviation of the AFL-CL histogram.

In line with prior mapping studies, the proportion of lines of CB was calculated as the number of lines of CB (interelectrode conduction time > 11 ms) relative to the total number of interelectrode connections. A cut-off value of >11 ms was derived from prior mapping studies in which this degree of delay was associated with reversal of wavefront direction at the other side of the line of CB. Beat-to-beat consistency in patterns of activation was evaluated by comparing entry sites of the AFL wave and main direction of propagation in consecutive wavemaps for each location. Frequency of fractionated potentials, defined as electrogram potentials consisting of 2 or more negative deflections, were quantified as the number of fractionated potentials relative to the total number of potentials. As we previously demonstrated that fractionated unipolar potentials are caused by remote, asynchronous activation of the atrial wall,^5^, ^6^, ^7^ all deflections of fractionated potentials were annotated in a separate analysis to identify electrical asynchrony in deeper tissue layers.

### Spatiotemporal variability of the atrial cycle length

Median AFL-CL ranged from 270 ms to 273 ms. The shortest (223 ms) and longest AFL-CL (321 ms) were both found at the superior part of the RA. AFL-CL variability per region ranged from 2 to 5 ms.

### Pattern of activation

**Figure 1:**
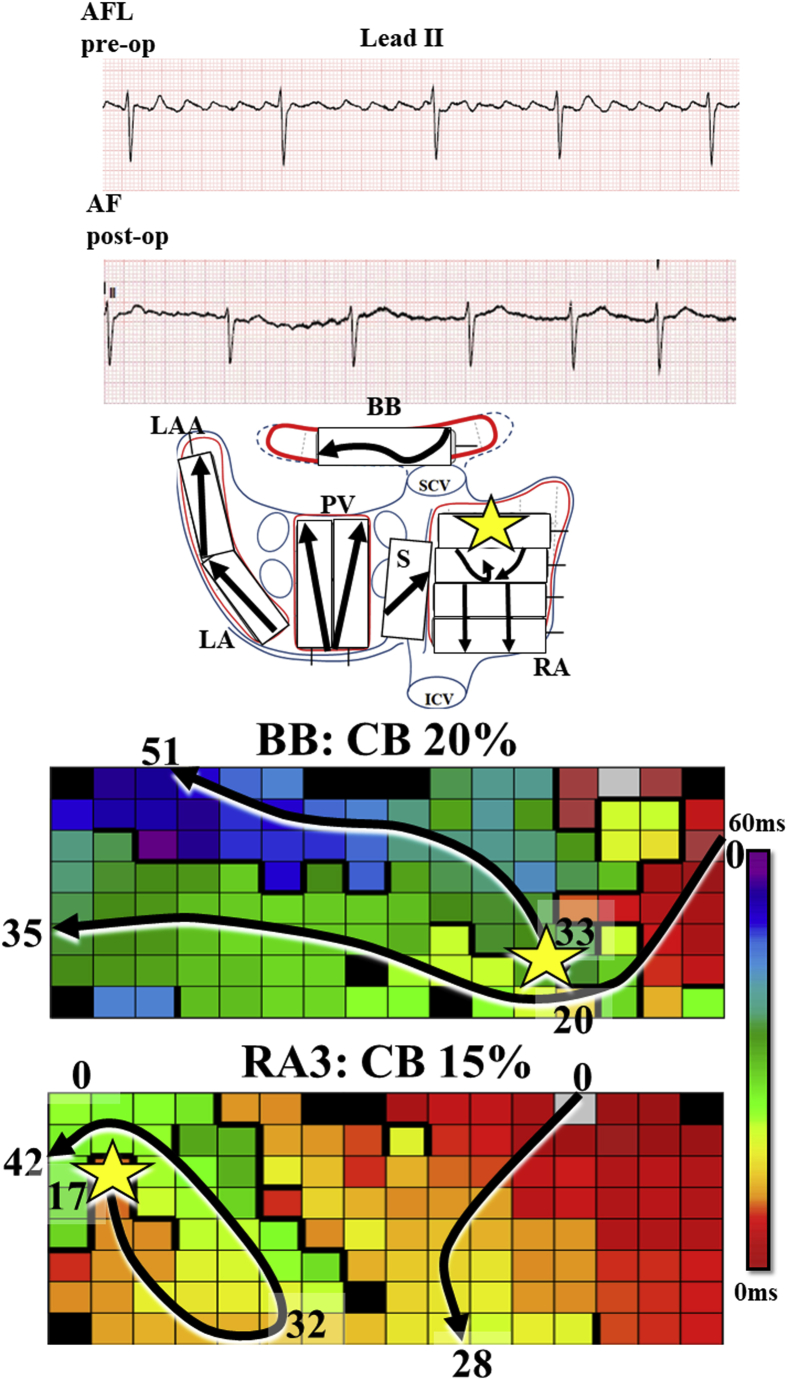
Activation pathway of atrial flutter (AFL). **Top:** Lead II of the preoperative surface electrocardiogram shows the typical saw-tooth wave pattern of a counter-clockwise AFL. The second electrocardiogram shows registration of early postoperative atrial fibrillation (AF). **Middle:** The main activation pathway during AFL with counter-clockwise rotation in the right atrium (RA) and passive activation of Bachmann's bundle (BB) and left atrium (LA). **Bottom:** The main wavefront during AFL trajectories (*arrow*) is affected by 20% and 15% conduction block (CB) (*thick black lines*) at BB and RA. Yellow stars indicate epicardial breakthrough waves. IVC = inferior vena cava; LAA = left atrial appendage; PV = pulmonary veins; S = septum; SCV = superior vena cava.

Lead II of the surface electrocardiograms depicted in the upper panel of Figure 1 shows the typical saw-tooth flutter waves that were present before and during cardiac surgery.

The arrows in the schematic presentation of both the RA and LA including BB in the middle panel of Figure 1 demonstrate direction of activation at all mapping locations. The main pattern of activation matches that of typical AFL, consisting of counter-clockwise activation of the RA and passive activation of BB and LA. This pattern of activation was consistent throughout the entire mapping procedure. Remarkably, both at the BB and the superior RA, the main pathway of the AFL wavefront is considerably curved.

Local activation time maps of these 2 sites are plotted in the lower panel. These maps show that the curvatures in wave trajectories were caused by long lines of CB, indicated by thick black lines. The proportion of CB lines is 20% at the BB and 15% at the high mid RA free wall. This is considerably higher compared to other atrial sites (5.1% at the posterior LA, 3.5% at the LAA, 0.2% at the LA apex, and 0.8%–4.5% at the remaining RA sites).

### Fractionated potentials

**Figure 2:**
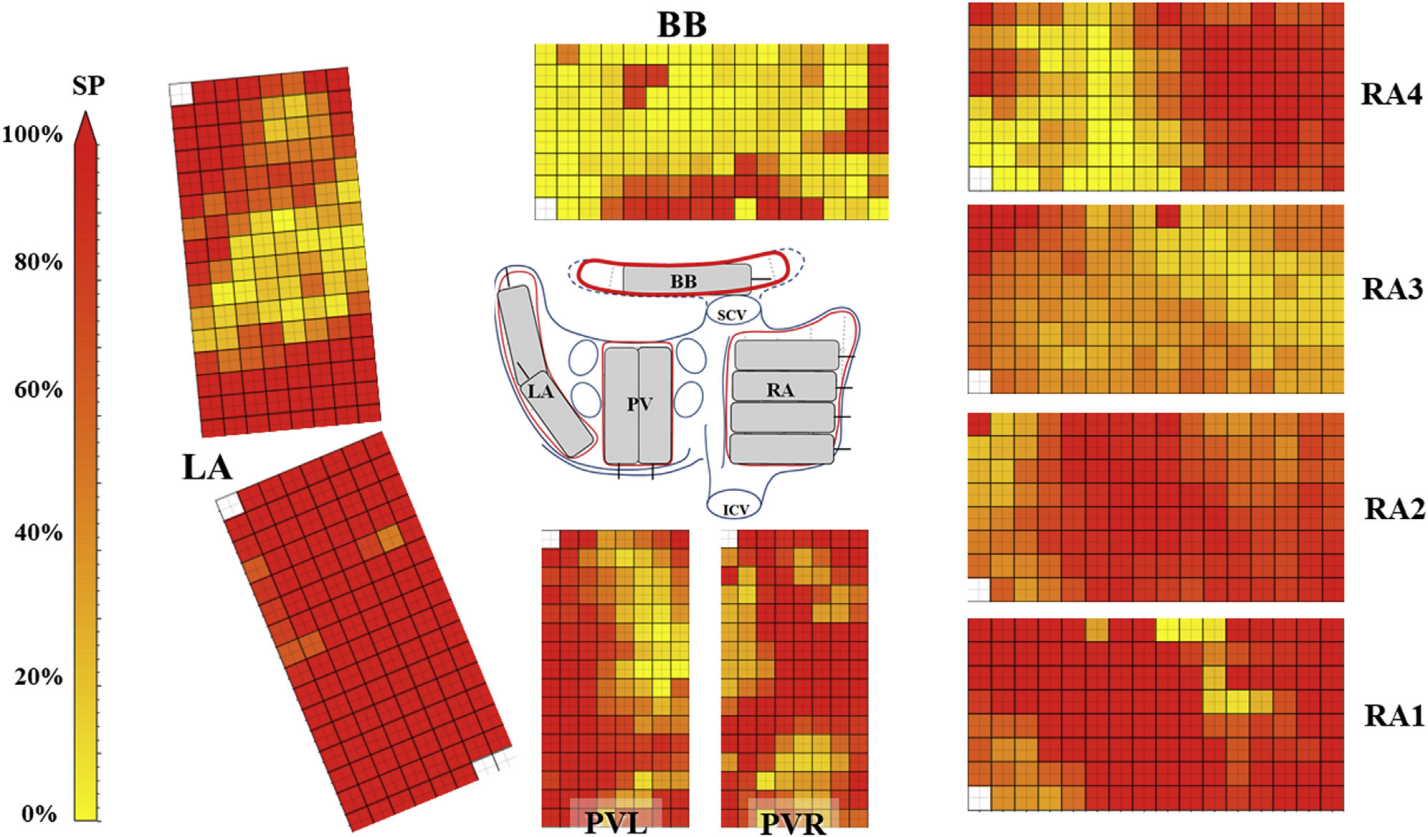
Unipolar electrocardiogram fractionation. Color-coded maps indicate the proportion of single potentials (SP) during 10-second recording. Areas with predominantly fractionated potentials are revealed at the Bachmann bundle (BB), the high right atrium (RA), and the left atrial appendage. ICV = inferior vena cava; LA = left atrium; PVL = left pulmonary vein; PVR = right pulmonary vein; S = septum; SCV = superior vena cava.

Figure 2 shows the frequency of single potentials at each electrode within every mapping location. Fractionated potentials predominantly occurred at the BB, the high RA, and the LAA. In contrast, from the inferior LA and lower RA mainly single deflections were recorded.

Patterns of activations of the BB and the high RA constructed by this mapping approach are shown in Supplemental Movie 1 and Supplemental Movie 2, respectively. At the BB (Supplemental Movie 1), the first wavefront enters from the lower right border (the RA side) and propagates towards the upper border of the array, following a slightly curved trajectory. After 32 ms, a second wavefront appears in the lower left corner, then zigzags towards the upper border. The outline of this second wavefront can be also recognized as the line of CB in Figure 1 and as a transition zone from predominantly single to fractionated potentials in Figure 2. Fractionation in this region was thus caused by a distinct, second wavefront propagating at deeper layers.

The pattern of activation at the high RA is even more complex, as multiple wavefronts propagate in opposite directions (Supplemental Movie 2). The right and left border of the mapping array are oriented towards the high and low RA, respectively. The first 2 wavefronts pass from the right to the left border, respectively, while the wavefront originating from the right border activates the majority of the mapping area. It propagates towards the left lower corner to activate the lower region for the second time. In the meantime, another wave enters from the upper left border and crosses towards the right border again, reactivating the upper part of the array. Annotation of fractionated potentials at this location thus also reveals the presence of different wavefronts activating tissue layers asynchronously; the very short delays (7–16 ms) between these consecutive wavefronts can, owing to atrial refractoriness, not represent reactivation of the same tissue. It is likely that electrical barriers between different strands of cardiomyocytes prevent transmurally asynchronous activation and give rise to different conduction corridors within the atrial wall.

## Discussion

Epicardial high-density mapping of a typical, counter-clockwise AFL revealed long lines of CB and fractionated U-EGM potentials. These conduction abnormalities resulted from remote waves propagating in deeper tissue layers, causing asynchronous activation of myocardial layers. To the best of our knowledge, this is the first report of indirect evidence for EEA during AFL.

### Evidence on interrelation between AFL and AF

The patient described in this case report developed AF after ablation for AFL, which is quite common. A strong interrelation between AF and AFL has already been recognized for a long time. Ellis and colleagues,^8^ for example, found that 82% of patients that underwent CTI ablation for typical AFL developed AF within a mean follow-up period of 39 ± 11 months. Although in a different study “only” 30% of patients developed AF after CTI ablation,^9^ a relation between the 2 tachyarrhythmias is likely.

The tachyarrhythmias not only coexist, but the onset of AF and AFL may even be interdependent, as induced AFL is commonly preceded by AF.^10^ The line of CB at the core of the reentrant circuit reaches its full length during high-rate excitation and stabilizes the tachyarrhythmia. When a reverse rhythm transition from AFL to AF takes place, the central line of CB shortens and multiple, migrating lines of CB are observed throughout the atria.^11^ Since the 2 arrhythmias frequently coexist and show interdependency, they may reflect a similar arrhythmogenic substrate. As previously proposed by Waldo and Feld,^12^ micro- and macroreentry may even be regarded as the same phenomenon on a different scale. In theory, when the reentrant circuit in AFL is sufficiently large and AFL-CL is long enough, the rest of the atria will passively follow in a 1:1 fashion. However, if the reentry circuit is too short, the atria are not able to conduct in a 1:1 manner, resulting in fibrillatory conduction. This mechanism is in line with theories of ectopic, high-frequency discharging foci or leading circle reentry during AF.

### Epi-endocardial asynchrony in AFL

EEA is a known indicator of the arrhythmogenic substrate during AF, as it occurs more often in patients with persistent AF than in patients without a history of AF.^13^ Evidence of EEA during AFL, however, is limited. In a recent case report, anatomically determined EEA was suspected in 2 patients with a roof-dependent AFL following endocardial PVI for persistent AF. In these patients, AFL could be entrained from within the box at the posterior LA wall during high-output pacing (50 mA/20 ms and 20 mA/10 ms) despite confirmed entrance and exit block (10 mA/2 ms). These observations suggested that the lesions were not transmural and an epicardial pathway was still part of the AFL circuit. The authors suggested that this epi-endocardial asynchrony was anatomically determined by the septopulmonary bundle stretched from the pulmonary veins to the superior side of the BB.^14^ However, spatial resolution of wave patterns was limited, as is inherent to endocardial mapping studies, which hampers precise interpretation of patterns of activation. Also, it cannot be excluded that pacing stimuli of 50 mA have entrained endocardial tissue from outside the box area.

In the present case, however, high-density epicardial mapping data enabled us to provide a detailed reconstruction of wave trajectories. In this way, propagation of multiple, short-coupled wavelets within the atrial refractory period exciting the same mapping area was visualized. These observations confirm that EEA can be present during AFL. EEA may be relevant to ablation therapy outcome, as it is likely that EEA reduces efficacy of ablation therapy when only 1 myocardial layer is targeted. EEA may also have predisposed the patient for AF onset by increasing complexity of the pre-existing arrhythmogenic substrate.

### Considerations

In the preoperative electrocardiogram, in addition to the typical saw-tooth AFL waves, upright AFL waves in lead V_1_ there were isoelectric AFL waves in lead I. Flat or isoelectric AFL waves in lead 1 suggest upper loop reentry, regardless of its clockwise or counter-clockwise orientation. However, flat AFL waves in lead I have also been described in patients with typical counter-clockwise AFL.^15^ Both AFL-CL and the total activation map showed no evidence of upper loop reentry. Therefore, AFL subtype in this patient was diagnosed as a typical counter-clockwise AFL.

## Conclusion

This case report demonstrates the presence of localized areas activated by different, consecutive wavefronts with very short delays (7–16 ms), suggestive of wavefronts propagating asynchronously in deeper layers of the atrial wall during a typical, counter-clockwise AFL. These observations provide indirect evidence of epi-endocardial asynchrony during AFL and may explain why this patient developed postoperative AF. Future research is warranted to investigate whether EEA underlies increased susceptibility to AF in patients with AFL.

## Appendix Supplementary data

Movie 1 Movie legends

Movie 2 Epi-endocardial asynchrony in BB At BB, the first wavefront enters from the lower right border (the RA side) and propagates towards the upper border of the array, following a slightly curved trajectory. After 32 ms, a second wavefront appears in the lower left corner, then zigzags towards the upper border. The outline of this second wavefront can be also recognized as the line of CB in Figure 1 and as a transition zone from predominantly single to fractionated potentials in Figure 2. Fractionation in this region was thus caused by a distinct, second wavefront propagating at deeper layers. Arrows indicate the main trajectory of wavefronts. BB=Bachmann’s bundle, CB=conduction block, LA=left atrium, RA=right atrium.

Movie Epi-endocardial asynchrony at the RA At the high RA, the first two wavefronts pass from the right to the left border, respectively, while the wavefront originating from the right border activates the majority of the mapping area. It propagates towards the left lower corner to activate the lower region for the second time. In the meantime, another wave enters from upper left border and crosses towards the right border again, reactivating the upper part of the array. Annotation of fractionated potentials at this location thus also reveals the presence of different wavefronts activating tissue layers asynchronously. Arrows indicate the main trajectory of wavefronts. RA=right atrium.
