# Supplementary material for: Epi-endocardial asynchrony during atrial flutter followed by atrial fibrillation
Source: HeartRhythm Case Rep. 2021 Jan 13;7(3):191–4. doi: 10.1016/j.hrcr.2021.01.001 (PMC7987922; doi:10.1016/j.hrcr.2021.01.001)
Supplement: Movie 1 — Movie legends [file mmc1.docx]

# Movie legends

Movie 1: Epi-endocardial asynchrony in BB

At BB, the first wavefront enters from the lower right border (the RA side) and propagates towards the upper border of the array, following a slightly curved trajectory. After 32 ms, a second wavefront appears in the lower left corner, then zigzags towards the upper border. The outline of this second wavefront can be also recognised as the line of CB in Figure 1 and as a transition zone from predominantly single to fractionated potentials in Figure 2. Fractionation in this region was thus caused by a distinct, second wavefront propagating at deeper layers. Arrows indicate the main trajectory of wavefronts. BB=Bachmann’s bundle, CB=conduction block, LA=left atrium, RA=right atrium.

Movie 2: Epi-endocardial asynchrony at the RA

At the high RA, the first two wavefronts pass from the right to the left border, respectively, while the wavefront originating from the right border activates the majority of the mapping area. It propagates towards the left lower corner to activate the lower region for the second time. In the meantime, another wave enters from upper left border and crosses towards the right border again, reactivating the upper part of the array. Annotation of fractionated potentials at this location thus also reveals the presence of different wavefronts activating tissue layers asynchronously. Arrows indicate the main trajectory of wavefronts. RA=right atrium.
